# The effectiveness and cost-effectiveness of spinal cord stimulation for refractory angina (RASCAL study): study protocol for a pilot randomized controlled trial

**DOI:** 10.1186/1745-6215-14-57

**Published:** 2013-02-22

**Authors:** Sam Eldabe, John Raphael, Simon Thomson, Andrea Manca, Mark de Belder, Rajesh Aggarwal, Matthew Banks, Morag Brookes, Susan Merotra, Rashidat Adeniba, Ed Davies, Rod S Taylor

**Affiliations:** 1 Department of Pain and Anesthesia, The James Cook University Hospital, Marton Road, Middlesbrough, UK; 2 Department of Pain Medicine, Dudley Group of Hospitals NHS Foundation Trust, Russells Hall Hospital, Pensnett Road, Dudley, West Midlands, UK; 3 Basildon and Thurrock University Hospitals, Nethermayne, Basildon, UK; 4 Centre for Health Economics, University of York, Heslington, York, UK; 5 Cardiothoracic Department, Plymouth Hospitals NHS Trust, Derriford Road, Plymouth, UK; 6 University of Exeter Medical School, University of Exeter, Veysey Building, Salmon Pool Lane, Exeter, EX2 4SG, UK

**Keywords:** Randomized controlled trial, Pilot study, Refractory angina, Spinal cord stimulation

## Abstract

**Background:**

The RASCAL (Refractory Angina Spinal Cord stimulation and usuAL care) pilot study seeks to assess the feasibility of a definitive trial to assess if addition of spinal cord stimulation (SCS) to usual care is clinically superior and more cost-effective than usual care alone in patients with refractory angina.

**Methods/design:**

This is an external pilot, patient-randomized controlled trial.

The study will take place at three centers in the United Kingdom - South Tees Hospitals NHS Foundation Trust (The James Cook University Hospital), Dudley Group of Hospitals NHS Foundation Trust, and Basildon and Thurrock University Hospitals NHS Foundation Trust.

The subjects will be 45 adults with refractory angina, that is, limiting angina despite optimal anti-angina therapy, Canadian Cardiovascular Society Functional Classification Class III and IV, angiographically documented coronary artery disease not suitable for revascularization, satisfactory multidisciplinary assessment and demonstrable ischemia on functional testing.

The study will be stratified by center, age and Canadian Cardiovascular Society Functional Classification.

Interventions will involve spinal cord stimulation plus usual care (‘SCS group’) or usual care alone (‘UC group’). Usual care received by both groups will include consideration of an education session with a pain consultant, trial of a transcutaneous electrical neurostimulation, serial thoracic sympathectomy and oral/systemic analgesics.

Expected outcomes will be recruitment and retention rates; reasons for agreeing/declining participation; variability in primary and secondary outcomes (to inform power calculations for a definitive trial); and completion rates of outcome measures. Trial patient-related outcomes include disease-specific and generic health-related quality of life, angina exercise capacity, intake of angina medications, frequency of angina attacks, complications and adverse events, and satisfaction.

**Discussion:**

The RASCAL pilot trial seeks to determine the feasibility and design of a definitive randomized controlled trial comparing the addition of spinal cord stimulation to usual care versus usual care alone for patients with refractory angina.

Fifteen patients have been recruited since recruitment opened in October 2011. The trial was originally scheduled to end in April 2013 but due to slow recruitment may have to be extended to late 2013.

**Trial registration:**

ISRCTN65254102

## Background

Refractory angina (RA) is defined by a European Society of Cardiology taskforce as a “clinical diagnosis based on the presence of symptoms of stable angina, thought to be caused by ischemia due to advanced coronary disease and which are not controllable by a combination of maximal medical therapy, bypass surgery and percutaneous intervention” [1]. Others have proposed that RA is an indication in which patients are refractory to drug therapy but also unsuitable for revascularization. The United Kingdom (UK) national chronic RA guideline group defines RA as “chronic stable angina that persists despite optimal medication and when revascularization is unfeasible or where the risks are unjustified” [2]. The most frequent reasons that patients are unsuitable for revascularization include unfavorable coronary anatomy, multiple previous revascularization procedures (both percutaneous and surgical), the lack of suitable bypass graft targets or conduits, and significant extra-cardiac co-morbidities [3]. It is estimated that in Europe the incidence of RA is 100,000 new cases per year [4]. RA patients experience severe chest pain, which can result in multiple hospitalizations and low levels of health-related quality of life [3]. A number of therapies have been recommended for the treatment of RA following optimization of their cardiovascular drug therapy, that is, beta blockers, nitrates, potassium agonists and newer generation drugs (for example, ranolazine) [1,3]. These additional therapies include sympathectomy, analgesics (usually opiate-based), angina management programs, stimulation induced analgesia, stellate ganglion block, epidural blocks, spinal cord stimulation (SCS), enhanced external counterpulsation and percutaneous laser myocardial revascularization [4,5].

The use of spinal cord stimulation (SCS) for angina was first described in 1987. SCS is a reversible procedure in which electrodes are implanted in the epidural space to stimulate the dorsal columns of the spinal cord [6]. The technique has been described in detail elsewhere [7]. SCS has been successfully used to relieve pain in a number of chronic conditions, including neuropathic pain and pain due to peripheral vascular disease [8].

The mechanism of action of SCS in neuropathic pain is well documented. For ischemic pain there are a number of proposed mechanisms. It is believed to have its effects by a combination of modulation of pain pathways within the central nervous system and anti-ischemic mechanisms. SCS modulates the autonomic nervous system, suppresses intrinsic cardiac nerve activity independent of beta sympathetic nervous system activity and is cardio-protective [9,10].

### Systematic review and meta-analysis

To summarize the current evidence for the use of SCS for RA, we updated a recent systematic review [11]. We searched a number of electronic databases including Medline, Embase and Cochrane Library up to April 2011 (with a further search in October 2012) to identify randomized controlled trials (RCTs) of SCS in RA. The reference lists of included studies were checked for potential additional studies. The search strategy, inclusion and exclusion criteria, study selection, data extraction and risk of bias assessment are detailed elsewhere [11].

We identified eight RCTs that included a total of 322 RA patients (see Additional file 1: Table S1) [12-19]. Five studies compared SCS stimulation (‘SCS ON’) to either sub-threshold or no SCS stimulation (‘SCS OFF’) [13,16-19], one study compared SCS to usual care [14], and two studies compared SCS with an alternative therapy (that is, coronary artery bypass graft [12] and percutaneous myocardial laser vascularization [13]). The risk of bias of five trials was judged to be high (that is, met two out of five criteria) and three trials to be low to moderate (that is, met three or more out of five criteria) (see Additional file 1: Table S2).

Findings are presented according to the two broad categories of control group, that is, SCS versus active intervention and active SCS versus no or sub-threshold SCS. The between group differences of active SCS (‘SCS ON’) versus no or inactive SCS (‘SCS OFF’) trials were quantitatively pooled using a conservative Der Simonian random effects meta-analysis model to take account of the potential heterogeneity (both clinical and methodological) across trials [20]. Given the variety of exercise capacity, consumption of nitrate medication and health-related quality of life outcomes were reported, results were expressed as a standardized mean difference (SMD). SMD is a summary statistic used when trials assess the same outcome, but measure it in a variety of ways [21]. The SMD expresses the size of the treatment effect in each trial relative to the study variance or standard deviation observed in the trial. All analyses were performed using RevMan, version 5.0 (http://www.cc-ims.net/RevMan).

Meta-analysis results are shown in Additional file 1: Figure S1. Outcomes of SCS were similar when directly compared to either coronary artery bypass grafting or percutaneous myocardial laser revascularization. Compared to a ‘no stimulation’ control, there was some evidence of improvement in all outcomes following SCS implantation with gains observed in pooled exercise capacity (SMD: 0.62, 0.03 to 1.21, Additional file 1: Figure S1a), short acting nitrate consumption (SMD: -0.65, 95%, -1.34 to 0.05, Additional file 1: Figure S1b), and health-related quality of life (SMD: 0.61, 95% CI: 0.13 to 1.09, Additional file 1: Figure S1c).

SCS-related complications were reported in five trials [12-14,16,18] and included infections (1 out of 104 patients, 1%) and lead migrations or fractures (10 out of 128 patients, 8%). Relatively few fatal and non-fatal events were reported and in no studies was there a statistically significant difference in events between SCS and the comparator [12,13,19].

### UK survey of current RA management

In designing a pragmatic trial of SCS for RA, it is important to determine what would constitute usual medical management and how this care might vary across centers in the UK.

It is likely that the management of patients with RA varies considerably. We were aware of only one UK specialist center for RA - the National Refractory Angina Centre in Liverpool. Outside of this center, the management of RA probably varies considerably by locality; some RA patients being managed within a cardiology unit, and others by a specialist pain team or a combination of the two. Furthermore, in the absence of authoritative evidence-based clinical guidelines for the management of RA, clinicians and centers may be recommending different therapies.

To assess current UK RA management and to quantify the potential variation in this practice, we undertook a national survey. Contact details were obtained for all UK pain centers (from the Dr Foster database) and cardiac centers (from the central cardiac audit database). After a number of rounds of drafting, we finalized a two-page questionnaire. A questionnaire and pre-paid return envelope were posted to all pain and cardiac units in April 2011. A follow-up questionnaire was posted to non-responding units in July 2011.

A total of 552 questionnaires was sent out and 215 replies were received (41%). Of these, 94 (44%) were from pain units, 49 (23%) were from cardiology units and the remainder (34%) did not indicate their specialty. The vast majority used either the European Society of Cardiology RA definition [1] (41%) or the UK National RA group definition [2] (54%) in their practice, the remainder (5%) stated that they used a different definition. Only 11 (5%) centers replied that they used a protocol or guideline for the management of their RA patients with RA. The drug therapies offered to patients with RA in addition to their cardiovascular drugs varied greatly with a total of 16 differing therapies identified. The most common treatments included regular slow release opiates (N = 122 units, 56%), instant release opiates as on demand prescriptions (101, 47%), transcutanous nerve stimulation (TENS) (116, 54%), exercise programs (84, 39%), cognitive behavioral therapy (72, 33%) and education (61, 28%). A total of 35 units (16%) used SCS. The complete list of RA treatments offered by units is summarized in Additional file 1: Figure S2.

Of the 192 (89%) centers who responded to the statement that ‘SCS was a suitable therapy for patients with RA’, 10% ‘strongly agreed’, 42% ‘agreed’, 42% were ‘unsure’, 5% ‘disagreed’ and 1% ‘strongly disagreed’. Of those who were unsure or disagreed, reasons given were that they did not know where to refer patients for SCS (26%), considered the clinical current evidence for SCS to be poor (46%), regarded SCS as too expensive (15%), did not understand how SCS worked (5%), were concerned that SCS may mask serious disease or felt RA did not require analgesia (1%). A total of 75 centers (44%) stated that they would be interested in participating in a multicenter trial involving patients with RA.

### Pilot study objectives

It is hypothesized that the addition of SCS to usual care will have superior clinical effectiveness and cost-effectiveness compared to usual care alone in patients with RA. The RASCAL (Refractory Angina Spinal Cord stimulation and usuAL care) pilot study seeks to assess the feasibility of a definitive trial to address this hypothesis. RA patients will be randomized to SCS plus usual care (‘SCS group’) or usual care alone (‘UC group’).

The objectives of this study are to:

i. To assess recruitment, uptake and retention of patients in both groups.

ii. To assess the feasibility and acceptability of SCS treatment from the point of view of patients and referring physicians.

iii. To assess the feasibility and acceptability of standardizing usual care from the point of view of patients and referring physicians.

iv. To test the feasibility and acceptability of the proposed trial outcome measures in both groups.

The results from this pilot study will inform the design and power for a definitive multicenter RCT.

## Methods/design

### Design

**Figure 1:**
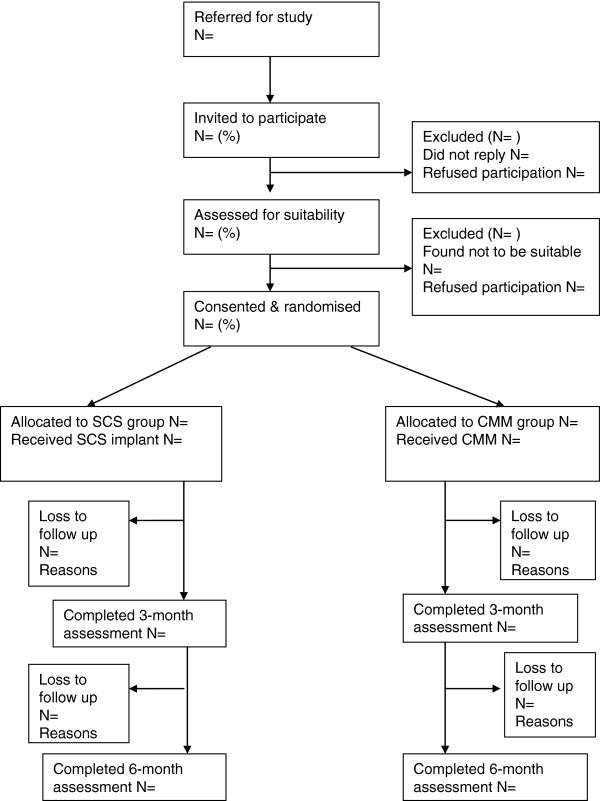
CONSORT flow for pilot trial.

This external pilot study is designed as a pragmatic multi-center study where RA patients are randomized in a 1:1 ratio to either the SCS or UC group. Given the paresthesia associated with SCS, it is not possible to blind patients, clinicians or researchers to group allocation. However, we will seek to blind those conducting the exercise capacity assessment. Research nurses conducting the data collection on the three sites will not be involved with patient care. The study design is summarized in Figure 1.

### Study population

Adults (≥18 years) with RA from South Tees Hospitals NHS Foundation Trust (The James Cook University Hospital), Dudley Group of Hospitals NHS Foundation Trust and Basildon and Thurrock University Hospitals NHS Foundation Trust will be invited to participate in the study.

#### Inclusion criteria

• limiting angina despite optimal anti-angina therapy

• Canadian Cardiovascular Society Functional Classification of Angina III and IV

• angiographically documented coronary artery disease

• coronary artery disease not suitable for revascularization in the opinion of the referring cardiologist/cardiothoracic surgeon

• satisfactory multidisciplinary assessment in accordance with British Pain Society guidelines for SCS [22]

• demonstrable ischemia on functional testing

#### Exclusion criteria

• presence of pacemaker or implanted defibrillator that is incompatible with SCS

• patient refusal to participate in the study

• presence of co-morbidity considered by the assessing clinician to overshadow the effect of the angina or render the patient an unsuitable candidate for neuromodulation (for example, advanced spinal disease or deformity)

• poor cognitive ability

• on-going anticoagulation therapy; where anticoagulants cannot be safely discontinued without jeopardizing patient safety

• patients who may require regular MRI scans

• pregnant or lactating females

• patients with special communication needs

### Recruitment of patients

Patients will be identified in the outpatient clinics of the cardiology departments of the three participating hospitals. Patients identified as suffering from RA will be discussed by the referring cardiologist with a colleague at consultant level to confirm the diagnosis. Following confirmation that the patient is suffering from RA they will be referred by letter, as well as, verbally to the pain clinic for treatment. Referred patients with a diagnosis of RA will have their notes reviewed by the research team to determine that they fulfill the trial inclusion criteria and will be invited to attend a multidisciplinary assessment in the pain clinic outpatient department.

Informed consent will be obtained from suitable patients by one of principal investigators (SE, JR or ST) or a delegated sub-investigator at each site. Patients will be provided with a patient information sheet and allowed a reasonable period of time to reflect upon their participation. A member of the research team will answer all questions regarding the study and risks of SCS before the patient signs an informed consent form.

### Sample size

A power calculation was not performed for this study to detect a clinically or economically meaningful difference in outcomes. Rather, the sample size was chosen to inform the feasibility objectives of this study, that is, provide estimates of the likely rates of recruitment and retention, and to yield estimates of the variability of the primary and secondary outcomes to inform power calculations for a subsequent full-scale RCT. We seek to randomize a total of 45 RA patients (that is, 15 patients/center).

### Randomization

Patients will be randomized sequentially, as they become eligible for randomization. The sequence of randomization will be computer-generated and stratified by hospital, age and Canadian Cardiovascular Society functional angina level. To ensure concealment, at the end of the screening visit the research nurse at each center will email the trial statistician (RST) with the relevant patient details, who will then allocate the patient. An email confirming the group allocation will be sent to the research nurse.

### Interventions

#### Spinal cord stimulation

For those patients allocated to receive SCS, the intervention will be started within six weeks from the date of randomization with an acute on-table trial of SCS followed by a final stage implant if the trial is successful.

SCS will be implanted by an experienced clinician (that is, one who has performed ≥15 SCS implants in the prior 12 months). The procedure will consist of inserting one or two epidural leads through a needle into a thoracic epidural interspace under local anesthetic with patients either in a prone lateral or sitting position according to patient and operator preference. Once in position, the leads will be connected to an external stimulator and the position adjusted to obtain optimum coverage of the painful area. In cases where the paresthesias cover 80% or more of the painful area the leads will be anchored to the spine via a small incision and connected via tunneled subcutaneous extensions, where required, to an implanted pulse generator placed in the anterior abdominal wall or the buttock according to patient’s and operator preference.

In case of failure to obtain >80% paresthesia coverage of the painful area, or if painful sensations are generated when the temporary stimulator is switched on, the procedure will be aborted, the leads removed and no pulse generator implanted (‘failed trial’). Patients who have a failed trial of SCS will continue to receive treatment and follow-up as per the study protocol.

Patients implanted with an SCS will be instructed how to adjust their device to generate comfort level paresthesias. They will be instructed to do this regularly for two hours three times daily, to terminate any angina attack for as long as is necessary, and before any exertion known or anticipated to generate angina pain. We will document usage of the SCS device through telemetry interrogation at the three- and six-month visits. The SCS devices to be used within this study are CE (Conformité Européene) marked and used within their intended license.

#### Usual care

Both groups of patients will receive the following combination of therapies started, if possible, on the day of randomization:

– education session with a pain consultant

– trial of a transcutaneous electrical nerve stimulation (TENS)

– serial thoracic sympathectomy

– oral/systemic analgesics and adjuvant analgesia

Patients who have already tried and failed to obtain relief from any of the sequence of therapies above will be moved on to the next therapy down the list if, in the judgment of the site principal investigator and the patient, the trial was considered to be adequate and well conducted. Following completion of the above sequence, the treating physician may apply any therapy deemed appropriate with the exception of repeat coronary artery bypass graft, percutaneous revascularization (or stenting) or percutaneous myocardial laser revascularization or enhanced external counterpulsation. We will document all UC care component treatments received by both groups of patients with start and end date where appropriate.

### Pilot study endpoints

The pilot study will collect and report on the following outcomes:

1. Recruitment and retention: we will document procedures for recruiting patients in both groups and any problems that arise during this process. Retention will be assessed by documenting the number of drop outs and lost to follow up in both groups.

2. Feasibility and acceptability of design: we will assess the time for attainment of patient number targets in each center, the proportion of suitable patients who fail to provide consent and the ratio of patients screened vs. randomized.

3. Feasibility and acceptability of treatment: patients will be asked to assess their willingness to recommend SCS or UC to other patients.

4. Feasibility and acceptability of proposed patient outcome measures: number of returned and complete outcome assessments assessed.

5. Outcome variance: mean and standard deviation for all outcomes will be calculated for each group at all assessment visits (to inform power calculations for a definitive trial).

### Trial outcome measures

Patient outcomes will be collected at clinic visits at baseline (pre-randomization), and three and six months post-randomization unless otherwise stated.

#### Primary outcome

Disease specific health-related quality of life will be assessed using the disease specific Seattle Angina Questionnaire (UK version) [23].

#### Secondary outcomes

– Intake of angina medications and frequency of angina attacks: We will supply patients with an angina diary to record the frequency of their angina attacks as well as the use of anti-angina medication. The average number of angina attacks per week will be recorded at baseline. Patients will be asked to fill the diary for one week prior to the three- and six-month visits. All patients will receive a phone call reminder from the research team asking them to start the diary. Any changes to cardiac medication will be noted during follow-up visits.

– Exercise capacity (at baseline and six months only) will be assessed by a symptom-limited treadmill (modified Bruce protocol). For each patient the same test will be undertaken at baseline and six months.

– Complications and adverse events: The nature and frequency of device-specific and non-device events by direct questioning of the patient and, where necessary, review of medical notes.

– Healthcare utilization (for example, cardiac specific hospitalizations and primary care visits, management of complications/adverse events) will be assessed at six months using the patient report. If necessary, the patient’s General Practitioner will be contacted by letter to seek these details. We will collect information on all current medication dosing and frequency at baseline and at the three- and six-month follow-ups.

– Generic health-related quality of life: Assessed using the EuroQol (EQ-5D) and Short Form-36 (SF-36) questionnaires [24,25].

– Satisfaction: At the six-month follow-up visit, patients will be asked to answer the following questions:

o Are you satisfied with the pain relief provided by your treatment?

(Very Satisfied/Satisfied/Unsatisfied/Very Unsatisfied)

o Based on your experience so far, would you have agreed to this treatment?

(Definitely Yes/Yes/No/Definitely No)

o Can you tell us how acceptable these treatments were to you? (a list of treatments will be provided)

(Extremely acceptable/Acceptable/Not acceptable/Extremely unacceptable /Not Applicable)

### Statistical analysis

Given the pilot nature of this trial, we do not propose to formally, statistically test differences in outcomes and costs between or within groups. Instead, for all groups we will estimate SCS and UC group mean and standard deviation (or frequency) for all outcomes at each assessment point. All analyses will be conducted using STATA v.11 (Stata Corporation, College Station, Texas, USA).

### Trial management and quality assurance

South Tees Hospitals NHS Foundation Trust will act as the lead sponsor for the trial. All research team members obtaining informed consent will have appropriate training and current Good Clinical Practice (GCP) certification. In line with the International Conference on Harmonization (ICH)/GCP, patients will be given a copy of their consent form to keep for reference; a further copy of the consent form will be filed in the patient’s health records. The original version will be filed in the Investigator Site File on site. A unique study label will be fixed to the front of the patient’s health records to indicate their participation in the study. We will contact the patient’s general practitioner by letter to inform them of the patient’s participation in the study.

The trial office will receive completed case report forms from the two other centrrs via the postal service as well as the site database. Upon receipt, data forms will be checked for completeness and merged into a master chart, which will be communicated to the study statistician. Patient confidentiality will be maintained at every stage and we will comply with the Data Protection Act (1998).

Patients may withdraw from the trial or the trial treatment at any time without prejudice. If a patient withdraws from the trial treatment, then they will be followed up wherever possible and data collected as per protocol until the end of the trial. The only exception to this is where the patient also explicitly withdraws consent for follow-up. Patients may be withdrawn from the study at the discretion of the Investigator/Trial Steering Committee due to safety reasons. South Tees Hospitals NHS Foundation Trust should be notified by faxing a notification of the patient withdrawal form within 48 hours of withdrawal. All adverse events and serious adverse events will be recorded at each visit and in between visits by the research nurses at each site on an adverse event log. The study coordinator will liaise with the study statistician on a regular basis and the Chief Investigator will be made aware of all adverse events and serious adverse events as they happen. In the event that the patient is withdrawn due to safety reasons, a serious adverse event or suspected unexpected serious adverse reaction notification should also be sent to the South Tees Hospitals NHS Foundation Trust Research and Development Department.

#### Trial steering committee

The trial will be overseen by the Trial Steering Committee (TSC). The TSC will consist of the chief investigator (SE), study coordinator (MB), site investigators (JR, ST, MdB), trial statistician (RST) and the following independent members:

Dr Duncan McNab (Chair) - Consultant cardiologist, Papworth Hospital NHS Foundation Trust and Mr David Richardson (lay member) - British Pain Society Patient Liaison Committee.

TSC meetings will be held not less than once a year. Routine business will be conducted by email, post or teleconferencing. In discussion with the TSC, it was agreed that a separate Data Monitoring Committee will not be necessary in this study. Instead, the independent TSC members will be presented with the safety data prior to meetings.

#### Trial management group

The trial administration will be overseen by a trial management group (TMG) chaired by the Chief Investigator (SE). Membership of the TMG includes: the principal investigator on all three trials sites (SE, JR, ST), study statistician (RST), study coordinator (MB) and study nurses (MB, RA and SM). The TMG will meet at six monthly intervals either face to face or by tele/video conferencing. The trial will be coordinated by the study coordinator (MB) based at The James Cook University Hospital. All day-to-day non-clinical coordination of the trial will be the responsibility of the trial coordinator. All clinical coordination of the trial will be the responsibility of the Chief Investigator. The Chief Investigator will assume responsibility for the overall management and conduct of the trial. Each principal investigator will assume responsibility for leading the trial in their center. The trial office team will distribute the clinical research forms to participating centers, monitor the collection of data, process data, seek missing data and clarify ambiguous data, ensure the confidentiality and security of all trial forms and data, and organize TSC meetings.

## Discussion

Our updated systematic review and meta-analysis shows that SCS appears to be an efficacious and safe treatment option in the management of RA. Nevertheless, that evidence is based on small trials of variable quality; our review supports the current American College of Cardiology/American Heart Association Grade ‘IIb evidence’ classification (that is, “conflicting evidence and/or divergence of opinion about the usefulness/efficacy of a procedure or treatment”) for SCS in RA patients [26]. Furthermore, the National Institute for Health and Clinical Excellence (NICE) has recently called for further high quality RCT evidence in order to confirm the clinical and cost-effectiveness evidence before SCS can be accepted as a routine treatment for RA in the UK [27]. That our national survey shows the variability in how UK centers currently manage their RA patients is challenging to the design of a pragmatic trial of SCS.

We have designed this pilot trial in order to test the feasibility of undertaking a definitive pragmatic RCT comparing the clinical and cost-effectiveness of the addition SCS to UC versus UC alone in RA patients. While we have attempted to standardize usual care, we will also record the variation (both within and between centers) in the usual care received by patients in this study. The results of this pilot will inform the design of a definitive RCT.

## Trial status

The RASCAL pilot trial began recruitment in October 2011. Fifteen patients have been recruited to date (1^st^ December 2012). The trial was originally scheduled to end in April 2013. However, due to slow recruitment, trial end may have to be extended to late 2013.

## Abbreviations

CE: Conformité Européene (European conformity); GCP: Good Clinical Practice; ICH: International Conference on Harmonization; NICE: National Iinstitute for Health and Clinical Excellence; RA: Refractory angina; RASCAL: Refractory Angina Spinal Cord stimulation and usuAL care; RCT: Randomized controlled trial; SCS: Spinal cord stimulation; SMD: Standardised mean difference; TENS: Trancutaneous electrical nerve stimulation; TSC: Trial steering committee; TMG: Trial management group; UC: Usual care.

## Competing interests

The authors wish to disclose the following competing interests: SE and RST have received consultancy fees from Medtronic and KW is an employee of Medtronic. The other authors have no conflicts.

## Authors’ contributions

SE is the trial Chief Investigator. RA, MB, SE, MdeB, AM, JR, RST and ST contributed to the concept and study design and funding acquisition. MB, RA and SM are responsible for patient recruitment and data collection. ED and RST drafted the first version of the manuscript. All authors have commented on drafts of the paper and have given final approval to this version.

## Supplementary Material

Additional file 1: Table S1 Systematic review – Characteristics of included trials. **Table S2.** Systematic review – Risk of bias of included trials. **Figure S1a.** Systematic review – Between group comparison in exercise-capacity. **Figure S1b.** Systematic review – Between group comparison in nitrate drug consumption. **Figure S1c.** Systematic review – Between group comparison in health-related quality of life. **Figure S2.** National survey – Frequency of RA therapy use across UK centers. Click here for file
