# Supplementary material for: The effectiveness and cost-effectiveness of spinal cord stimulation for refractory angina (RASCAL study): study protocol for a pilot randomized controlled trial
Source: Trials. 2013 Feb 22;14:57. doi: 10.1186/1745-6215-14-57 (PMC3598727; doi:10.1186/1745-6215-14-57)
Supplement: Additional file 1: Table S1 — Systematic review – Characteristics of included trials. Table S2. Systematic review – Risk of bias of included trials. Figure S1a. Systematic review – Between group comparison in exercise-capacity. Figure S1b. Systematic review – Between group comparison in nitrate drug consumption. Figure S1c. Systematic review – Between group comparison in health-related quality of life. Figure S2. National survey – Frequency of RA therapy use across UK centers. [file 1745-6215-14-57-S1.docx]

**e-Table 1. Systematic review – Characteristics of included trials**

| **First author/trial name (year)**  **Country** | **Total number of RA patients** | **Intervention** | **Comparator** | **Primary outcome** | **Follow up** |
| --- | --- | --- | --- | --- | --- |
| De Jongste (1994) Netherlands | 22 | SCS | No SCS | Exercise capacity | 2-months |
| DiPede (2001)  Italy | 30 | SCS ON | SCS OFF | Ischemic burden | 48-hours |
| EBSY (1998)  Sweden | 104 | SCS | Coronary artery bypass graft | Exercise capacity, angina symptoms | 6-month, 2-years, 5-years |
| Hauvast (1998) Netherlands | 25 | SCS ON | SCS OFF | Exercise capacity | 1.5-months |
| Jessurum (1999) Netherlands | 24 | SCS ON | SCS OFF | Exercise capacity | 1-months |
| SpiRiT (2006)  UK | 68 | SCS | Percutaneous laser myocardial revascularisation | Exercise capacity | 3, 6, 12, 14-months |
| Eddicks (2007) Germany | 24 | SCS ON | SCS OFF | Exercise capacity | 1-month |
| Lanza (2011)  Italy | 25 | SCS ON | SCS OFF | Angina episodes | 1,3,6 & 12-months |

**e-Table 2. Systematic review – Risk of bias of included trials**

|  | **Random sequence generation** | **Sequence concealment** | **Blinding** | **Loss to follow up/drop out (%)** | **Methods of data analysis** | **Risk of bias score+** | **Funding** |
| --- | --- | --- | --- | --- | --- | --- | --- |
| De Jongste (1994) | Not stated | Independent telephone service | Open label | 8% | Not stated | 2/5 | Government |
| DiPede (2001) | Not stated | Not stated | Blinded ECG assessment | 0% | Not stated | 2/5 | Not stated |
| EBSY (1998) | Not stated | Not stated | Open label | 7%* | Intention to treat | 2/5 | Government |
| Hauvast (1998) | Not stated | Not stated | Open label | 0% | Not stated | 1/5 | Government |
| Jessurum (1999) | Not stated | Not stated | Open label | 0% | Not stated | 1/5 | Industry |
| SpiRiT (2006) | Computer generated | Independent R&D department | Open label | 9% | Intention to treat | 4/5 | Industry |
| Eddicks (2007) | Not stated | Not stated | Double blind | 0% | Not stated | 2/5 | Industry |
| Lanza (2011) | Not stated | Not stated | Single blind - patients | 0% | Intention to treat | 3/5 | Industry |

*: at 6-months; +: number of criteria fulfilled

**eFigure 1a. Systematic review – Between group comparison in exercise-capacity**


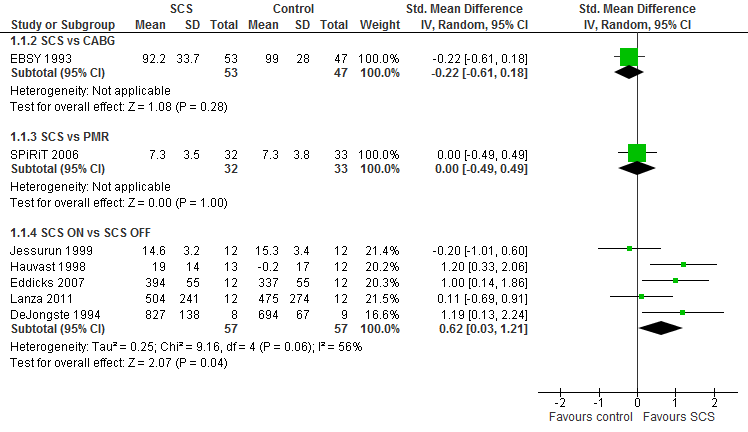


**eFigure 1b. Systematic review – Between group comparison in nitrate drug consumption**


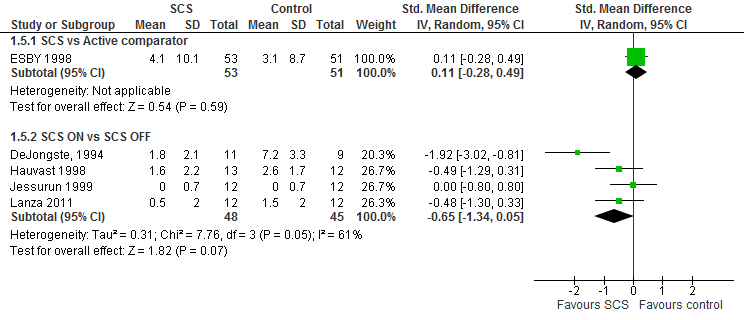


**eFigure 1c. Systematic review – Between group comparison in health-related quality of life**


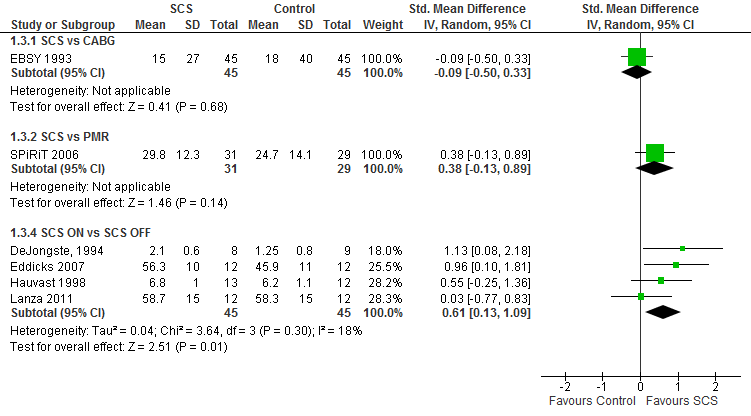


**eFigure 2. National survey – Frequency of RA therapy use across UK centres**
